# The Role of Estrogen Receptors on Spatial Learning and Memory in CA1 Region of Adult Male Rat Hippocampus 

**Published:** 2010

**Authors:** Anis Talebi, Naser Naghdi, Hori Sepehri, Amaneh Rezayof

**Affiliations:** a *Faculty of Biology, College of Sciences, Tehran University, Tehran, Iran. *; b *Department of Physiology and Pharmacology, Pasteur Institute of Iran, Pasteur Avenue, 13164 Tehran, Iran.*

**Keywords:** Hippocampus, CA1, Estrogen receptor, Tamoxifen, PPT, Spatial learning and memory

## Abstract

The hippocampal system plays an important role in memory function. Neurohormones like androgens and estrogens that syntheses in hippocampus have an important role in learning and memory. Many biological effects of estrogens in the brain via estrogenic receptors (ERs) are investigated. The current research has conducted to assess the effect of estrogenic receptors on spatial discrimination in rats by using Morris water maze (MWM) task. Adult male rats were bilaterally cannulated into CA1 region of hippocampus and divided in to 9 groups. Different groups received dimethyl sulfoxide (DMSO) 0.5 μl as control groups and different doses of tamoxifen (TAX) as antagonist of ER (0.0625, 0.125 and 0.25μg/0.5μl), propyl pyrazol thiol (PPT) as agonist of ERα (5,10 and 20μg/0.5μl), TAX 0.25μg/0.5μl + PPT 20μg/0.5μl all days 30-35 min before training. Our results have shown TAX (0.25μg/0.5μl), PPT (20μg/0.5μl), TAX (0.25μg/0.5μl) + PPT (20μg/0.5μl) groups significantly increase the escape latency and traveled distance to find invisible platform. Our results indicate that TAX and PPT and also TAX (0.25μg/0.5μl) + PPT (20μg/0.5μl) impaired acquisition of spatial learning and memory. As a consequence, it seems that estrogen modulates memory function via a novel estrogenic mechanism.

## Introduction

The involvement of brain regions in spatial learning and memory may be considerably more complex and may comprise a large number of regions and pathways than originally proposed (1). Some evidences indicate that the hippocampus is necessary for acquisition and retrieval of spatial information as well as for consolidation/storage (2-5). This structure is known to be a target for the neuromodulatory actions of hormones produced in the gonads as well as in the brain. Recent experiments have demonstrated that hippocampal neurons are exposed to locally synthesized brain steroids (4,6,7). Some observations suggest that hippocampal neurons are equipped with a set of enzymes to catalyze the synthesis of testosterone and estrogen from cholesterol (3,8). The basal concentration of estradiol was approximately 0.006 pmol/mg protein (600 pm), which is roughly six times greater than the plasma level (3). This information serves an excellent model for the study of physiological roles of neurosteroids in the brain, because hippocampal neurons play an important role in learning and memory (7). It is shown nuerohormones influence brain development, cognition, memory, and behavior and these effects are mediated by steroid hormone receptors (9-13). Several reports suggest that in addition to the well-known traditional effects of androgens and estrogens via intracellular receptors (genomic receptors), there are nongenomic androgen receptors that they have activational effects that maybe coupled to membrane ion channels and second messenger systems, which elicit rapid and transient changes in neuronal excitability (3,13-16). 

Some researchers suggest that there are a high density of estrogen receptors in fundamental centers of learning and memory in brain, such as the hippocampus (12,15,17,18). It is shown that there must be some relationship between estrogen receptors and cognitive aspects in the CNS (3,15,18).

The first member of what is now known as the steroid and thyroid hormone receptor superfamily was a protein isolated from the rat uterus exhibiting specificity for 17β-estradiol (9). This protein remained the only estrogen receptor (ER) known in animal tissues, until 1996 when a second ER subtype was isolated from the rat prostrate and ovary (19). Regarding these findings, there are two ER isoforms described: ER α and ER β (12,20-22). Because ER β is coded by a gene located on a different chromosome than the ERα gene, the two ERs are not true isoforms (9). Estrogen exerts a variety of effects on many regions of the nervous system via estrogenic receptors that influence higher cognitive functions. Some studies had shown that treating the primary hippocampal neurons with additional estradiol resulted in upregulation of ERα and downregulation of ERβ (12), therefore estradiol can act via ERα on learning and memory in CNS. Also, Fugger et al. (2000) showed a role for ERα in establishing cognitive function in an inhibitory avoidance task (20). OVX ERαKO mice exhibit retention deficits as measured by reduced latencies on Day 2. This impairment was not observed for ERβKO mice suggesting an important role for ERα in the establishment of cognitive processes involved in inhibitory avoidance performance (20,23). Furthermore, ERα activation impairs acquisition of spatial learning in Morris water maze (23). Other studies have shown that ERβ is an important modulator of cell proliferation and learning and memory (20,24) that had previously been postulated for other areas of the brain (25). According to the different controversial results and finding that was mentioned, in this study, our aim is to distinguish proyal pyrazol thiol (PPT) (an agonist of ERα) and tamoxifen (TAX) (an antagonist of ER) microinjection in to CA1 region of hippocampus of adult male rat on spatial learning and memory in MWM.

## Materials and Methods

*Subject*

Male albino Wistar rats (200–250 g) obtained from Pasteur Institute of Iran were used throughout the study. Rats were housed three per cage, but one day before testing they were housed one per cage and were maintained at 25±2 ◦C under standard 12:12 h light–dark cycle with light on at 07:00 a.m. Food and water were available *ad libidum*. All experiments with animals were carried out in accordance with recommendations from the Declaration of Helsinki and internationally accepted principles for the use of experimental animals.

*Surgery*

Sixty three adult male rats were divided into 7 groups with 9 rats in each group. Rats were anesthetized with ketamine and xylazine (100 mg/kg and 25 mg/kg i.p.) and placed in a Stereotaxic instrument (Stoelting, USA). Bilateral guided cannula’s were implanted in the right and left CA1 and were attached to the skull surface using dental cement and jewelers screws. Stereotaxic coordinates based on Parxinos and Watsons atlas of the rat brain were: anterior–posterior (AP), −3.8mm from bregma; medial–lateral (ML), ±2.2mm from midline; and dorsal–ventral (DV), −2.7mm from the skull surface.

*Microinjection procedure*

Intracereberal injection was made through guided cannula (21-gage) using injection needles (27-gauge) connected to 0.5μl Hamilton micro-syringes by polyethylene tubing. The injection needle was inserted 0.3mm beyond the tip of the cannula. Then, 0.05μl of the vehicle (dimethyl sulfoxide, DMSO), tamoxifen (Tax), propyl pyrazol thiol (PPT) were injected into each side of CA1 region over 2 min and the left in place for an additional 60 s to allow for diffusion away from the needle tip. All injections were done 30–35 min before testing each day. In other experiment, we were injecting simoltaneuosly tow drugs in the brain of rats.

*Behavioral assessment*

*Apparatus*

The water maze is a black circular tank 136 cm in diameter and 60 cm in height. The tank was filled with water (20±1^◦^C) to a depth of 25 cm. The maze was located in a room containing many extra maze cues (e.g. bookshelves, refrigerator and poster). The maze was divided geographically into four quadrant (Northeast (NE), Northwest (NW), Southeast (SE), Southwest (SW)) and starting positions (North (N), South (S), West (W), East (E)) that were equally spaced around the perimeter of the tank. A hidden circular platform (diameter: 10 cm) was located in the center of the SW quadrant, submerged 1 cm below the surface of water. A video camera was mounted directly above of maze to record rats swim path. A tracking system was used to measure the escape latency, traveled distance and swimming speed of each rat and also the percent of distance and the time in each quadrant

*Procedure*

All testing began at 8:00 a.m. Each rat placed in the water facing the wall of the tank at one of the four designated starting points and allowed to swim and find the hidden platform located in the SW quadrant of the maze on every trial. The single training session consisted of eight trials with different starting positions that were equally distributed around the perimeter of the maze. Starting points were varied in a quasi-random fashion so that in each trialthe subject started from each location once and never started from the same place on any day. During each trial, each rat was given 90 s to find the hidden platform. If rat found the platform it was allowed to remain on it for 30 s and then, was placed in a holding cage for 30 s until the start of the next trial. After completion of training, the animals returned to their home cages until the retention testing (probe trial) 24 h later. In the probe trial the hidden platform was removed and the animal was released from the north location and allowed to swim freely for 60 s. After the probe trial, the platform elevated above the water surface and placed in the different position (SE quadrant). 

*Histology*

Following behavioral testing, animals were sacrificed by decapitation and brains were removed. For histological examination of cannula and needle placement in CA1 area, 100-μm thick section was taken, mounted on slides. Stained with cresyl violet and the cannula track were examined for each rat. Only those animals whose cannulas were exactly placed in CA1 region were used for data analysis.

## Experimental

*Experiment 1*

The aim of this experiment was to determine the effect of intrahippocampal injection of TAX as an antagonist of estrogenic receptor on MWM performance. A total of 24 rats divided into three groups according to the dose levels: 0.25, 0.125, 0.0625μg of TAX dissolved in 0.5μl DMSO.

*Experiment 2*

The aim of this experiment was to determine the effect of intrahippocampal PPT, as an agonist of ERα on MWM performance. A total of 27 rats divided into three groups according to the dose levels: 5, 10, 20μg PPT dissolved in 0.5μl DMSO.

*Experiment 3*

The aim of this experiment was to determine the effect of intrahippocampal injection of PPT plus TAX as an agonist of ERα and an antagonist of estrogenic receptors, respectively on MWM performance of 7 rats in one group according to the dose levels: 20μg + 0.25μg for PPT and Tax, respectively, dissolved in 0.5μl for each dose.

*Statistical analysis*

Statistical evaluation was done by Kolmogorov–Smimov test at first, to examine the normal distribution by using SPSS software. Data obtained over training days from hidden platform tests and in visible platform tests were analyzed by t-test for comparison between two groups and one-way analysis of variance (ANOVA) followed by Tukey’s test for multiple comparisons. All results were shown as means S.E.M. In all statistical comparisons, P < 0.05 was used as the criterion for statistical significance.

## Results

*Experiment1: the effect of Tamoxifen, TAX (antagonist of ERs)*

*Hidden platform trails*

**Figure 1:**
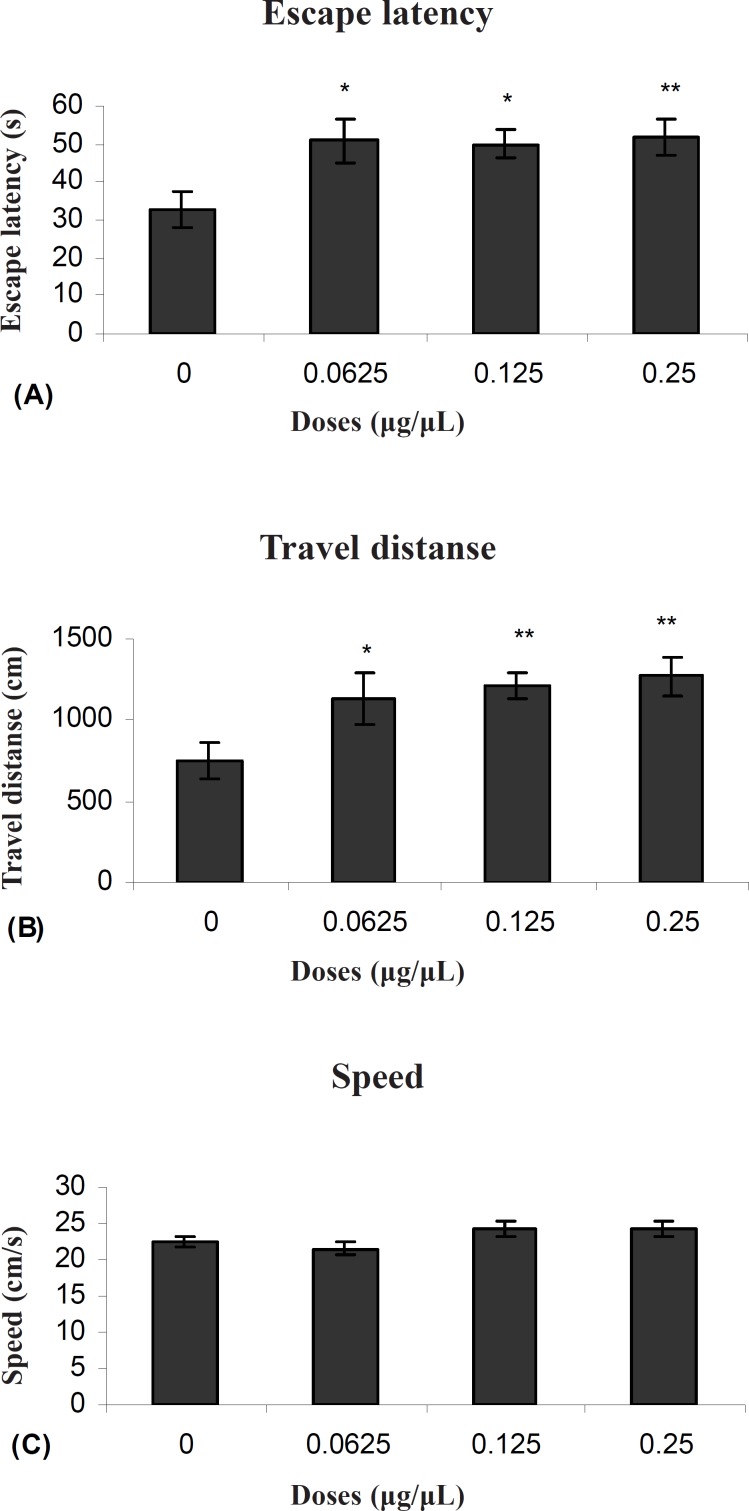
Escape latency (A), average traveled distance (B) and swimming speed (C) across all training. Figures show a significant difference in traveled distance. **P < 0.003 and escape latency **P < 0.005 between 0.25μg/0.5μl tamoxifen treated group with the control group

Figure 1 (A and B) shows the results obtained from injection of TAX compared to the group receiving DMSO as control. A significant difference was generally found in escape latency (F (3, 27) = 3071) and traveled distances (F (3, 27) = 3.83) between the groups. But no significant differences were found in swimming speed (F (3, 27) = 2.11) between the groups (Figure 1C).

*Probe test*

**Figure 2:**
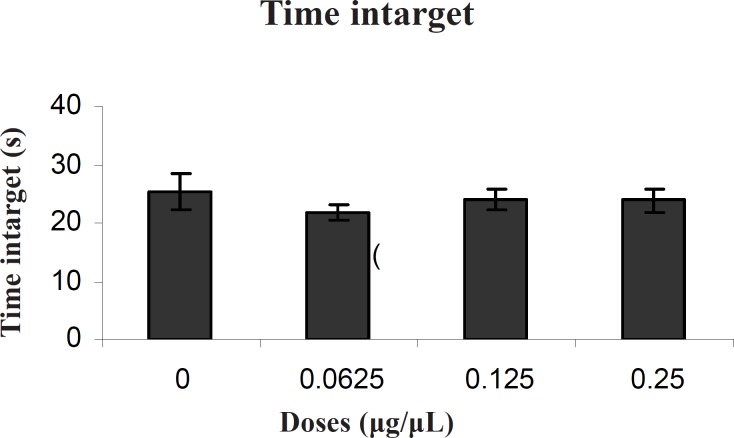
Comparison of time intarget during probe test within different groups. In probe test, there were no significant differences in time intarget

There was no significant difference in performance among the groups on the probe test for time intarget (F (3, 27) = 0.50) (Figure 2).

*Experiment 2: the effect of propyl pyrazol thiol, PPT (agonist of ERα)*

*Hidden platform trials*

**Figure 3:**
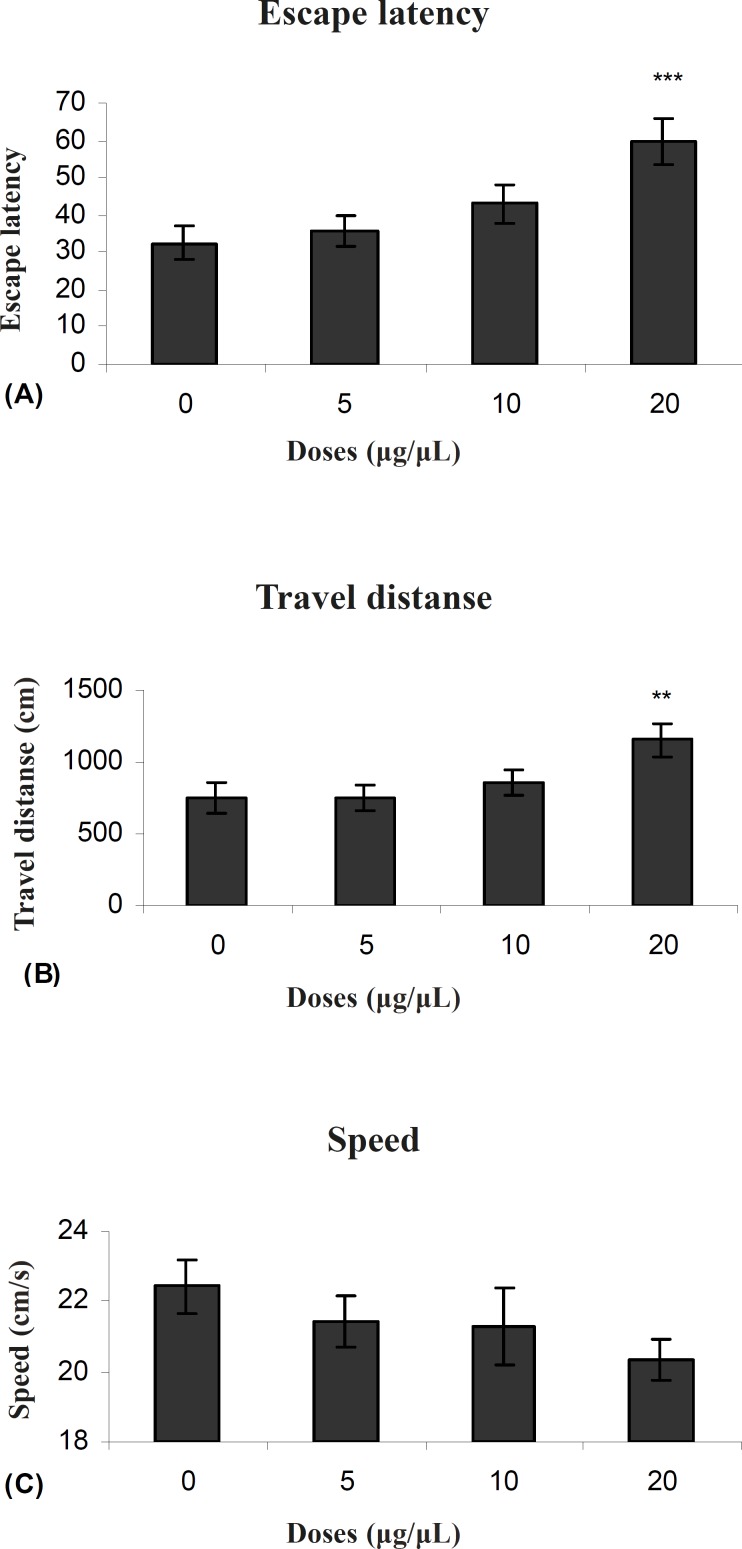
Escape latency (A), average traveled distance (B) and swimming speed (C) across all training. Figures show a significant difference in traveled distance **P < 0.007 and escape latency ***P < 0.000 between 20 μg/0.5μl propyl pyrazol thiol treated group with the control group

Figure 3 (A and B) shows the results obtained from injection of PPT and the group receiving DMSO as control. A significant difference was generally found only in 20μg/0.5μl group in escape latency (F (3, 27) = 5.911) and traveled distances (F (3, 27) = 3.714) between groups. But no significant differences were observed in swimming speed (F (3, 27) = 1.1192) between groups (Figure 3C).

*Probe test*

**Figure 4:**
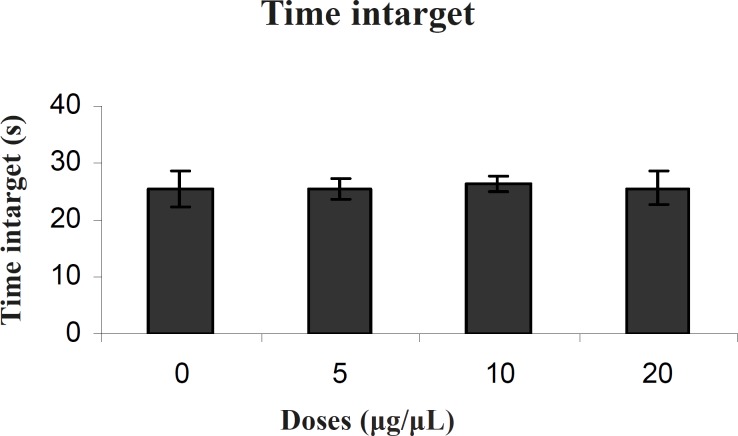
Comparison of time intarget during probe test within different groups. In probe test there were no significant differences in time intarget

There was no significant difference in performance among the groups on the probe test for time intarget (F (3, 27) = 0.021) (Figure 4).

*Experiment 3: the effect of TAX (antagonist of ERs) and PPT (agonist of ERα)*

*Hidden platform trials*

**Figure 5:**
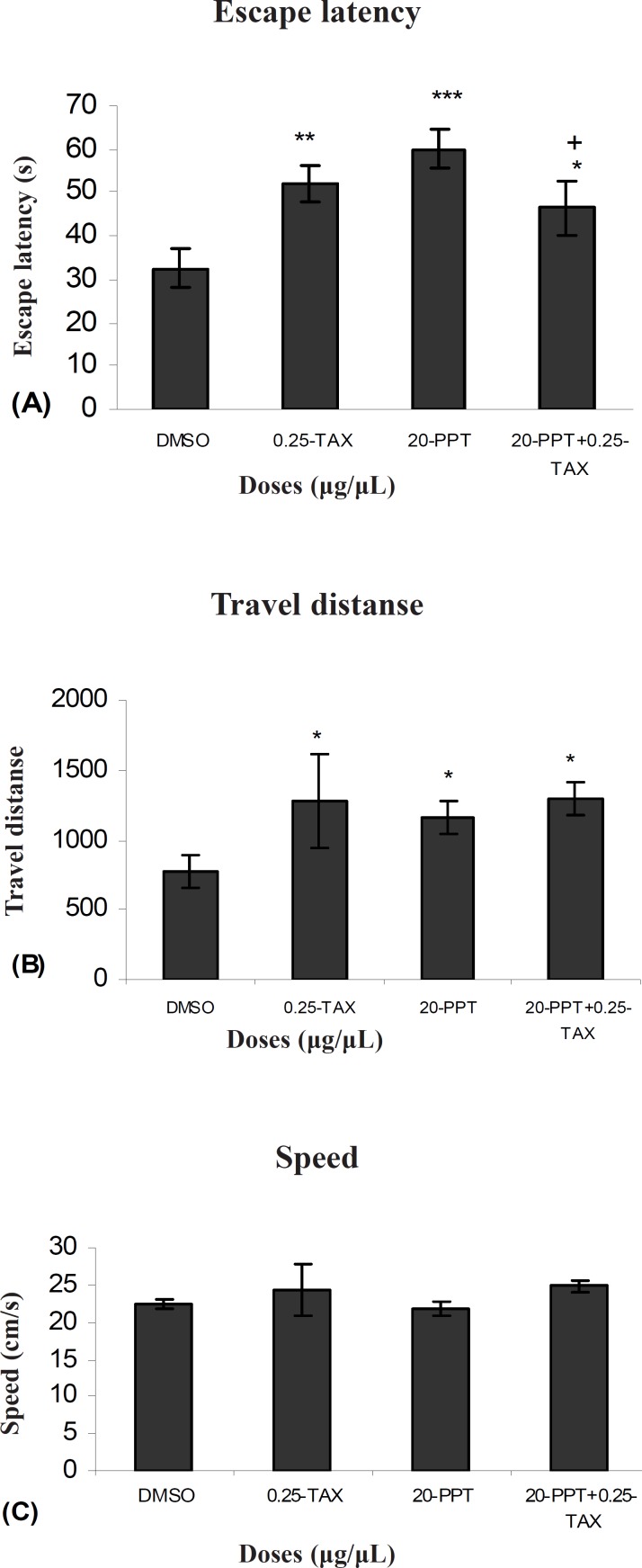
Escape latency (A), average traveled distance (B) and swimming speed (C) across all training. Figures show a significant difference in traveled distance *P < 0.065 and escape latency *P < 0.03 between TAX (0.25μg/μl) + PPT (20μg/μl) treated group with the control group. Also show a significant difference in traveled distance+ P <0.047 between TAX (0.25μg/μl) + PPT (20μg/μl) treated group with PPT (20μg/μl) treated group

Figure 5 shows the results obtained from injection of TAX and after 5–7 min injection PPT to determine the effect of an antagonist of ERs in presence of an agonist of ERα compared to the group received DMSO (control). A significant difference was generally found in escape latency (F (1, 9) = 0.55) between TAX + PPT treated group with the control group. (Figure 5 A). But no significant differences were found in swimming speed (F (1, 9) = 1.81) and traveled distances (F (1, 9) = 1.10) between them. ( Figure 5 B and C). A significant difference was found in escape latency (F (1,9) = 0.71) between TAX and PPT treated group with PPT treated group (Figure 5A). But no significant differences were observed in swimming speed (F (1, 9) = 1.43) and traveled distances (F (1, 9) ) between them (Figure 8B and C). Also no significant differences were found in swimming speed (F (1,9) = 1.99) and travel distance (F (1,9) = 0.81) and escape latency (F (1,9) = 1.02) between TAX+PPT with TAX group.

*Probe test*

**Figure 6:**
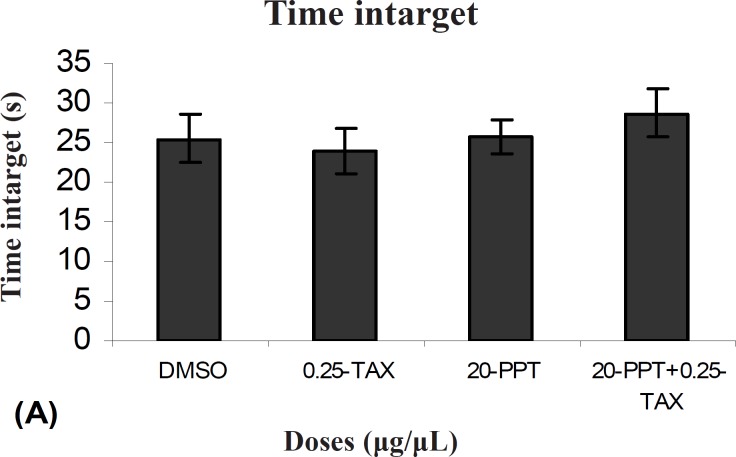
Comparison of time intarget during probe test within different groups. In probe test there were no significant differences in time intarget

There was no significant difference in performance between TAX+PPT treated group with the control group on the probe test for time intarget (F (1, 9) =0.331). Also there was no significant difference in performance between TAX + PPT treated group with PPT treated group (F (1, 9) = 1054) and between TAX + PPT treated group with TAX treated group (F (1, 9) = 0.92) (Figure 6).

## Discussion

Our results showed that TAX (0.25 μg/0.5μl) as an ERs antagonist and PPT (20μg/0.5μl) as an ERα selective agonist significantly increased escape latency and travel distance in comparison to control group.

These results showed that PPT and TAX could impair acquisition of memory in water maze task. Also the results showed that TAX (0.25μg/0.5μl) could eliminate PPT (20μg/0.5μl) - induced impairment. In all experiments, there were no significant differences in swim speed indicating that there were no significant differences in sensory or motivational processes between the groups.

The mechanism of action of TAX on impairing learning and memory functions has not been clearly evaluated (26). Estrogen replacement induces a 30% rise in both NMDA receptors and spines in the hippocampus of ovariectomized female rats (26). Thus, one may speculate that TAX could affect the function of hippocampus to impair the memory ability (27). Because TAX is an estrogen receptor antagonist in the central nervous system (26), it could be assumed that this activity is the main cause of their memory impairing action. 

A close interaction between estrogen and cholinergic function in the central nervous system has been reported (28-30). It has been inferred that estrogen affects learning and memory behaviors by modulation of basal cholinergic function (27). For example, it is reported that steroidal sexual hormones can affect on acetylcholine transferase and acetylcholine esterase activities. The long-term memory is facilitated with estradiol in the one-trial passive avoidance conditioning test in adult male Wistar rats (31). In ovariectomized rats, high-affinity choline uptake is reduced in the hippocampus and in the frontal cortex by 24% and 34%, respectively. This decline in high-affinity choline uptake is associated with a significant decrease in total avoidance in the active avoidance tests. Estrogen administration could reverse the effects of ovariectomy on high-affinity choline uptake in active avoidance and spatial memory behavior (32). These data suggest that cholinergic neurons are estrogen-responsive and that continuous exposure to ovarian steroid is needed to maintain the normal memory function. Since TAX is an antiestrogenic drug, it is reasonable to assume that TAX - induced impairment memory might be due to the blockade of the estrogen actions, which subsequently affects the activity of the cholinergic system (28).

Other mechanisms of action of TAX on memory function could not be excluded. It has been shown that TAX has a wide variety of pharmacological activities, such as the inhibition of protein kinase C (33), acting as a calmodulin antagonist (34), and acting as a histamine antagonist (35). These TAX actions may directly or indirectly affect on memory function (27).

Our results also showed that PPT (20 μg/0.5 μl) as an ERα selective agonist impaired acquisition of memory in Morris Water Maze task. Thus, it is possible that estrogen impairs memory via ERα. There are two possibilities for this finding: 

1- Regulation of spinogenesis is one of the important estrogen roles in memory processes via producing new spines for creating new neuronal contacts (36). Dendritic spine morphology analysis demonstrated that the density of thin type spines was selectively increased in CA1 pyramidal neurons within 2 h after application of 1 nM estradiol (21).

Sometimes the spine density is decreased by the estradiol treatment. The estradiol-induced spinogenesis is region specific and heterogeneous. In fact, in CA3 pyramidal neurons, the total density of thorns of thorny excrescences (spine-like postsynaptic structures in the stratum lucidum of CA3, having contacts with mossy fiber terminals originated from granule cells) decreased dramatically to approximately 70% upon a 2-h treatment of 1 nM estradiol (37). PPT significantly decreased the density of thorns from 2.19 to1.66 thorns/μm. Therefore, it may be PPT-induce impair memory processes by decreasing of spine density.

2- In memory processing, LTP, long term potentiation, (memory forming mechanism) and LTD (long term depreciation) are essential. Mutant mice, which show enhanced LTP and suppressed LTD, have shown impaired learning of Morris water maze (38). This suggests that LTD may be required to “correct” wrong memories formed by initial LTP processes, which store not only correct information but also wrong information. (21,39). Investigations using specific estrogen agonists indicated that the contribution of ERα (but not ERβ) was essential to these estradiol effects. PPT at 100 nM exhibited a significant LTD enhancement in CA1 (39). Therefore, it may be suggested that PPT impaired learning and memory via LTD enhancement and derangements in “correct” wrong memories formed by initial LTP processes.

In addition, our study showed that TAX as ERs antagonist (0.25 μg/0.5μl) could impair acquisition in MWM task. Also, our results showed that using TAX (ERs antagonist, 0.25 μg/0.5 μl) with ERα selective agonist (PPT (20 μg/0.5 μl) ) could eliminate impairment of using of PPT. It is demonstrated that estrogen modulates memory function via estrogenic receptors, especially ERα. Because PPT (agonist of ERα) in present of TAX (antagonist ERs) also shows an impairment effect on spatial learning and memory. Thus, it is possible that TAX has higher affinity to bind ERα than ERβ. Fugger N. et al. (2000) showed that ovariectomized (OVX) estrogen receptor-α-knockout (ERαKO) mice displayed impaired performance on the inhibitory avoidance (IV) task. In contrast to ERαKO mice, IA performance by OVX estrogen receptor-β-knockout (ERβKO) mice was not compromised. The results indicate an important role for ERα, relative to ERβ, in the establishment of cognitive function (20). Only the estrogen receptor (ER) α-agonist, propyl pyrazole trinyl tris-phenol (PPT), induced the same enhancing effect as estradiol on spinogenesis in the CA1. The ERbeta agonist, (4-hydroxyphenyl)-propionitrile (DPN), did not affect on spinogenesis (21). Regulation of spinogenesis is one of the important estrogen roles in memory processes via producing new spines for creating new neuronal contacts (36). It demonstrates that probably ERα is more important than ERβ in learning and memory process. Other studies have shown that ERβ is an important modulator for learning and memory (9,24). Considering downregulation of ERα in the hippocampus and amygdalohippocampal area in estrogen-treated knockout individuals, it was proposed that ERβ influenced spatial memory by binding estradiol and preventing this downregulation, a mechanism that had previously been postulated for other areas of the brain (9). Therefore, one could say that there are many of unknown proceeding in basis of learning and memory, also involvement of estrogenic receptors types in this process. 

In summery, it seems that intra hippocampal injections of TAX as ERs antagonist and PPT as ERα selective agonist could impair spatial learning and memory in MWM task. Also results showed that TAX could eliminate impairment of memory by injection of PPT. This finding suggests that estrogens act in learning and memory via ERs, specifically ERα. In this process many different mechanisms involved such as, distinct differences in binding and transactivation properties of α- and β-receptors, interaction between estrogen and cholinergic function in CNS, spinogenesis, LTP (long term potentiation), LTD (long term depreciation), ERs particularly ERα .
